# Supplementary material for: Label-Free Imaging to Track Reprogramming of Human Somatic Cells
Source: GEN Biotechnol. 2022 Apr 20;1(2):176–91. doi: 10.1089/genbio.2022.0001 (PMC9092522; doi:10.1089/genbio.2022.0001)
Supplement: Supplemental data [file Supp_FigS1.docx]

**
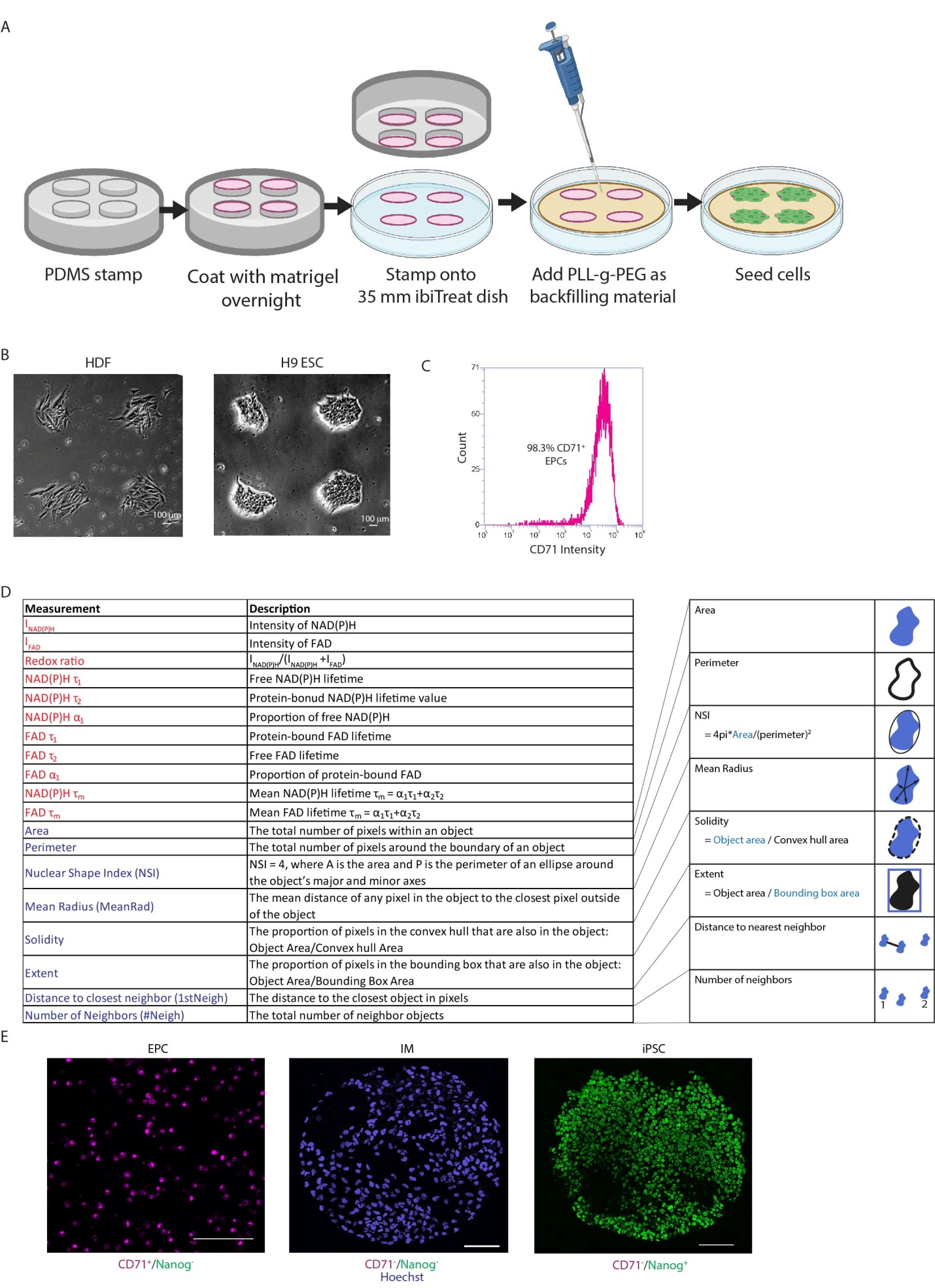
**

**Fig. S1. Micropatterned substrates enable controlled cell adhesion. A)** Schematic showing the fabrication of µCP substrates. PDMS mold is coated with Matrigel and stamped onto a 35 mm ibiTreat dish. PLL-g-PEG solution is then added to backfill the non-printed regions. Fabricated micropatterned substrates are then ready to be seeded with cells. **B)** Representative images of HDFs and H9 ESCs adhered to 300 µm radius circular µFeatures on micropatterned substrates. Scale bar, 100 µm. **C)** Flow cytometry histogram indicating the percentage of CD71^+^ cells after 10 days of EPC culture and before electroporation with reprogramming plasmids. **D)** Description of the 11 metabolic and 8 nuclear parameters that were measured. These parameters were obtained after processing NAD(P)H and FAD images using an image analysis pipeline described in Figure 1B. **E)** Representative images of cells on μFeatures stained with CD71(magenta), Hoechst (blue) and Nanog (green). EPCs are identified as CD71^+^; Nanog^-^, IMs are indicated as CD71^-^; Nanog^-^, and iPSCs are indicated as CD71^-^; Nanog^+^. Scale bar, 100 µm.
