# Supplementary material for: Label-Free Imaging to Track Reprogramming of Human Somatic Cells
Source: GEN Biotechnol. 2022 Apr 20;1(2):176–91. doi: 10.1089/genbio.2022.0001 (PMC9092522; doi:10.1089/genbio.2022.0001)
Supplement: Supplemental data [file Supp_FigS2.docx]

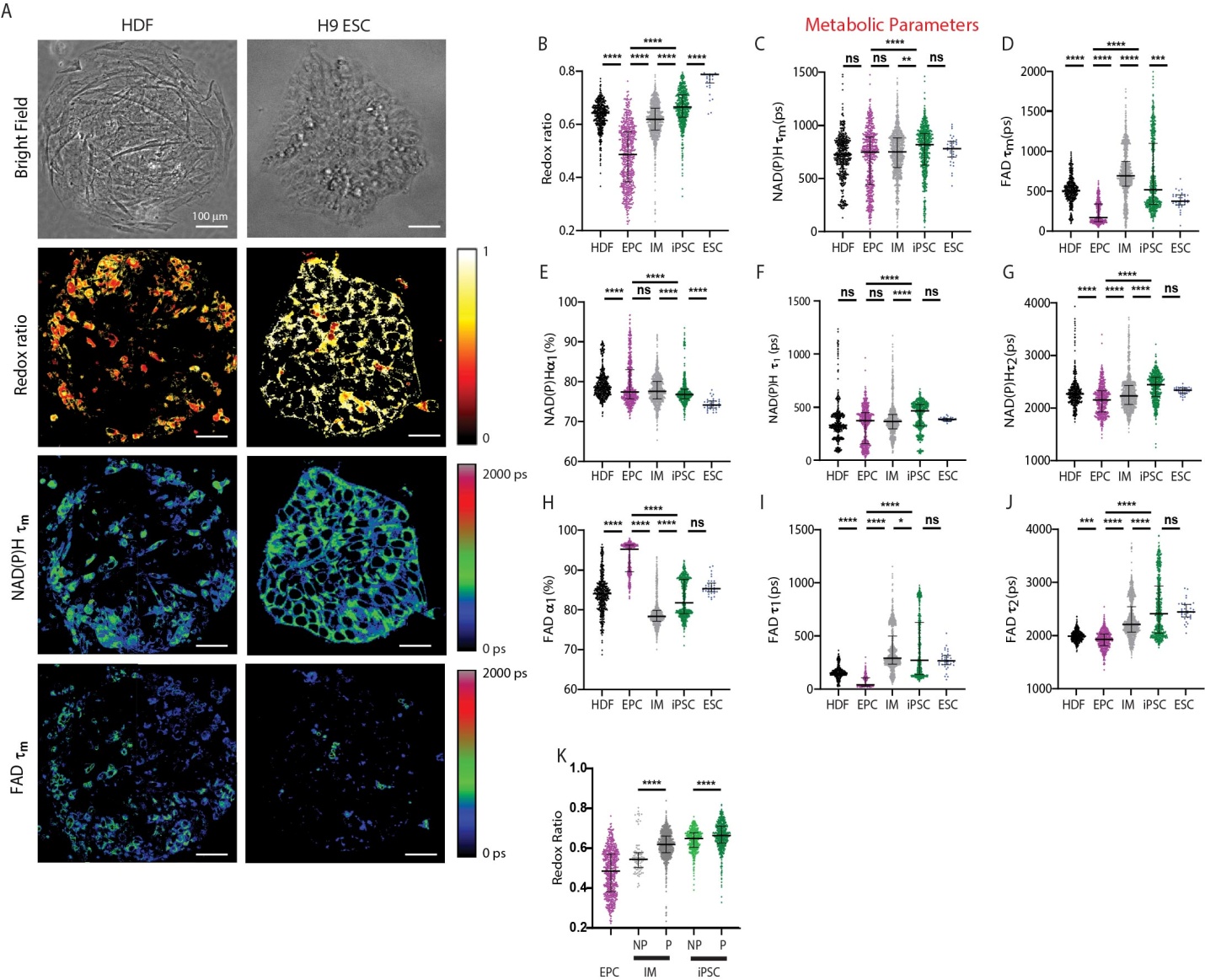


**Fig. S2. Metabolic parameter changes during reprogramming of EPCs. A)** Representative optical redox ratio, NAD(P)H τ_m_ and FAD τ_m_ images for HDFs and H9 ESC. Color bars are indicated on the right. Scale bar, 100 µm. Quantitative analysis of **B)** optical redox ratio [I_NAD(P)H_/(I_FAD_+I_NAD(P)H_)], **C)** NAD(P)H τ_m_, **D)** FAD τ_m_, **E)** NAD(P)H α_1,_ **F)** NAD(P)H τ_1_, **G)** NAD(P)H τ_2_, **H)** FAD α_1_, **I)** FAD τ_1_, and **J)** FAD τ_2_ for HDFs, EPCs, IMs, iPSCs and H9 ESCs at the single-cell level (n = 459, 561, 990, 586, 35 respectively). **K)** Quantitative analysis of optical redox ratio for non-patterned (NP) and patterned (P) reprogramming cells (IMs and iPSCs) at the single-cell level. Data are presented as median with interquartile range for each cell type. Statistical significance was determined by one-way analysis of variance (ANOVA) using the Kruskal-Wallis test for multiple comparisons; ns(non-significant) for p ≥0.05, * for p <0.05, ** for p <0.01, *** for p <0.001, **** for p <0.0001).
