# Supplementary material for: Label-Free Imaging to Track Reprogramming of Human Somatic Cells
Source: GEN Biotechnol. 2022 Apr 20;1(2):176–91. doi: 10.1089/genbio.2022.0001 (PMC9092522; doi:10.1089/genbio.2022.0001)
Supplement: Supplemental data [file Supp_FigS3.docx]

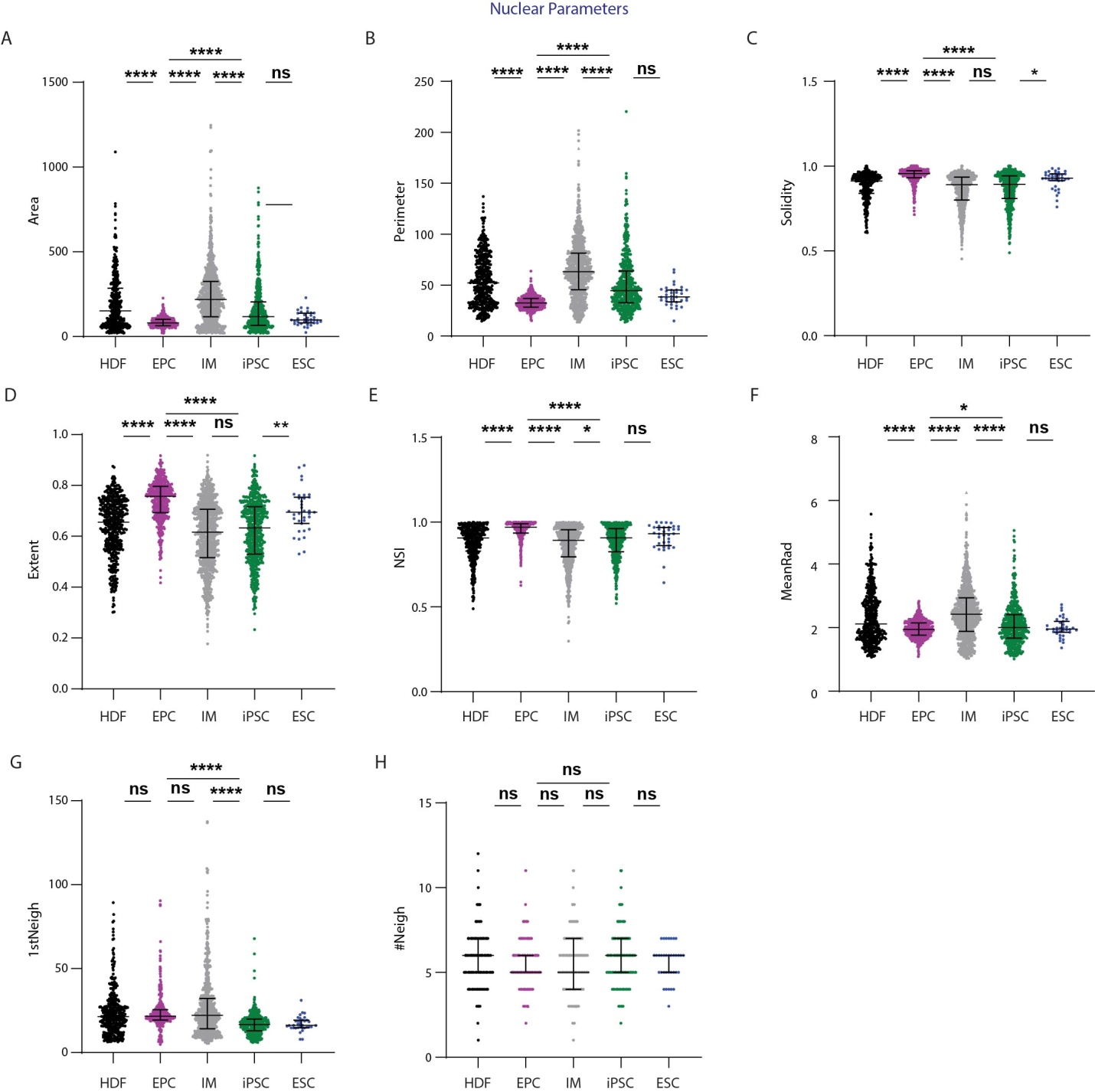


**Fig. S3. Nuclear parameter changes during reprogramming of EPCs.** Quantitative analysis of **A)** Area, **B)** Perimeter, **C)** Solidity, **D)** Extent, **E) Nuclear Shape Index (**NSI)_,_ **F)** Mean Radius **(**MeanRad), **G)** Distance to closest neighbor **(**1stNeigh), and **H)** Number of neighbors (#Neigh) for HDFs, EPCs, IMs, iPSCs and H9 ESCs at the single-cell level (n = 459, 561, 990, 586, 35 respectively). Data are presented as median with interquartile range for each cell type. Statistical significance was determined by one-way analysis of variance (ANOVA) using the Kruskal-Wallis test for multiple comparisons; ns(non-significant) for p ≥0.05, * for p <0.05, ** for p <0.01, *** for p <0.001, **** for p <0.0001).
