# Supplementary material for: Label-Free Imaging to Track Reprogramming of Human Somatic Cells
Source: GEN Biotechnol. 2022 Apr 20;1(2):176–91. doi: 10.1089/genbio.2022.0001 (PMC9092522; doi:10.1089/genbio.2022.0001)
Supplement: Supplemental data [file Supp_FigS4.docx]

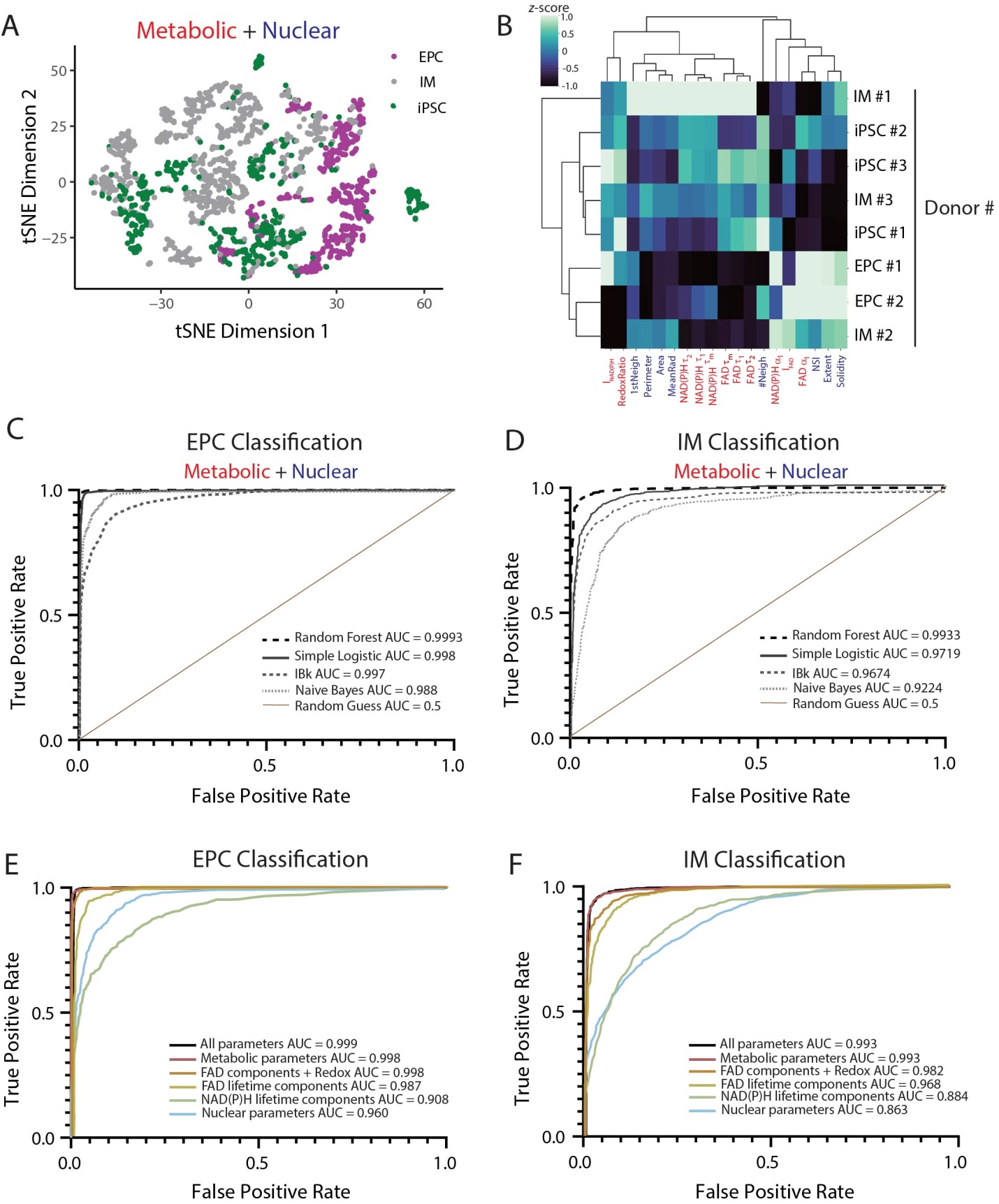


**Fig. S4. OMI enables accurate classification of EPCs, IMs, and iPSCs. A)** t-distributed stochastic neighbor embedding (t-SNE) dimensionality reduction was performed on all 11 metabolic and 8 nuclear parameters for each cell, projected onto 2D space, and shows poor separation of different cell types (EPCs, IMs, and iPSCs). Each color corresponds to a different cell type. Data are from three different donors. Each dot represents a single cell, and n =561, 990, and 586 cells for EPCs, IMs, and iPSCs, respectively. **B)** Heat map of *z*-scores (*z*-score = (𝜇_𝑜𝑏𝑠𝑒𝑟𝑣𝑒𝑑_−𝜇_𝑟𝑜𝑤_)/σ_𝑟𝑜𝑤_, where *μ_observed_* is the mean value of each parameter for a cell type; *μ_row_* is the mean value of each parameter for all cells together, and *σ_row_* is the standard deviation of each parameter across all cells. ) of metabolic and nuclear parameters; each row is the mean data aggregating all cells from a single donor and cell type (EPCs, IMs, iPSCs); n = 3 biologically independent donors. ROC curves for **C)** EPCs and, **D)** IMs for different classifiers computed using all 11 metabolic and 8 nuclear parameters. AUC is provided for each classifier as indicated in the legend. ROC curves for **E)** EPCs and, **F)** IMs for different classifiers computed using different parameter combinations. AUC is provided for each parameter combination as indicated in the legend.
