# Supplementary material for: Label-Free Imaging to Track Reprogramming of Human Somatic Cells
Source: GEN Biotechnol. 2022 Apr 20;1(2):176–91. doi: 10.1089/genbio.2022.0001 (PMC9092522; doi:10.1089/genbio.2022.0001)
Supplement: Supplemental data [file Supp_FigS5.docx]

**
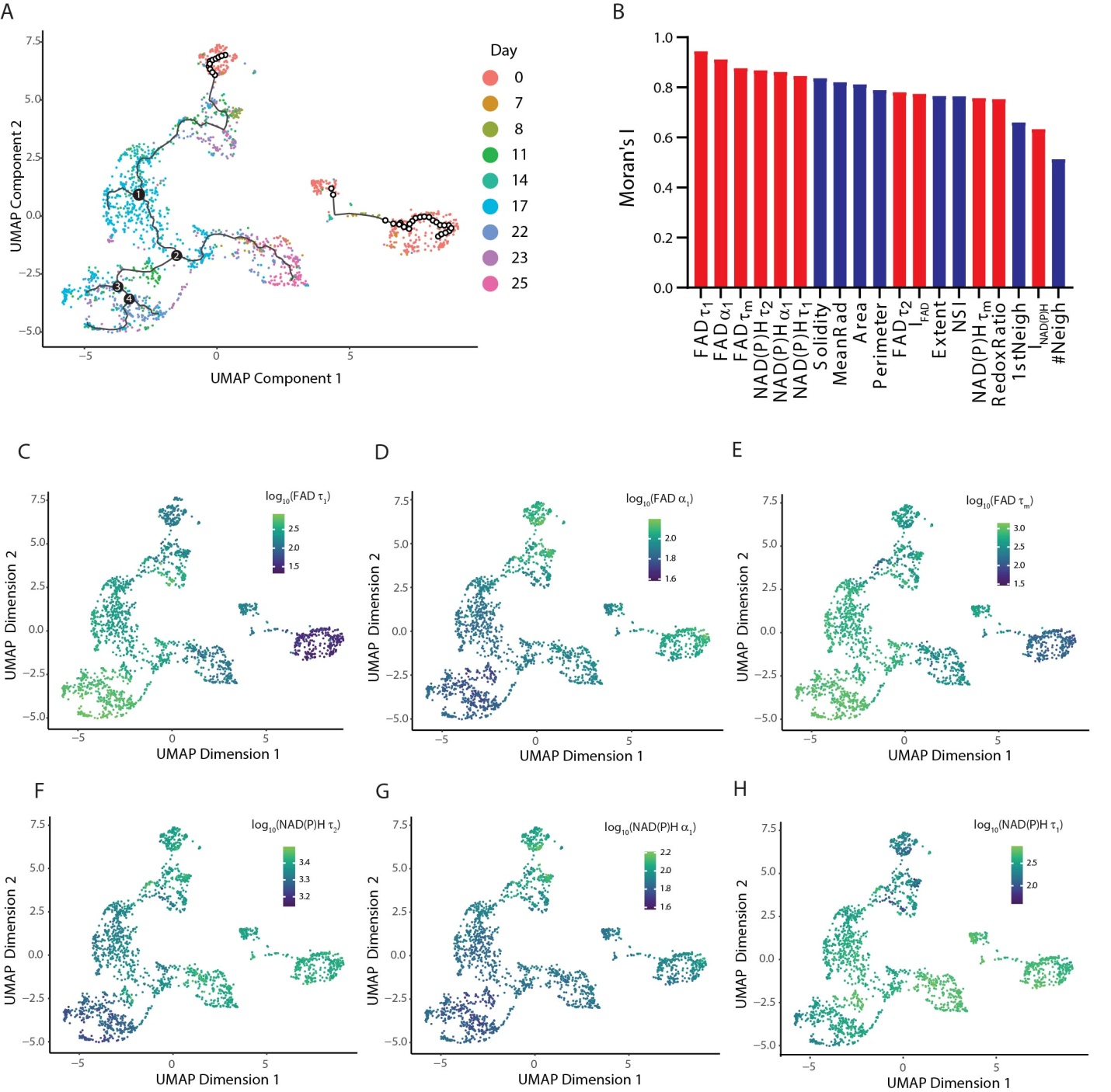
**

**Fig. S5. Metabolic and nuclear parameter changes along the reprogramming trajectory. A)** Trajectory of reprogramming EPCs constructed from the metabolic and nuclear parameters based on UMAP dimension reduction using Monocle, colored by day of EPC reprogramming. **B)** Metabolic and nuclear parameters ranked by their Moran’s I on the construction of reprogramming trajectory at the single-cell level. Moran’s I value of 1 means that nearby cells will have perfect correlation, 0 represents no correlation, and -1 means that neighboring cells will be anti-correlated) UMAP plots based on Figure 3A highlighting the change of expression of top-six metabolic parameters **C)** FAD τ_1_, **D)** FAD α_1_, **E)** FAD τ_m_, **F)** NAD(P)H τ_2_, **G)** NAD(P)H α_1_ and, **H)** NAD(P)H τ_2_ during reprogramming.
